# Restricted kinematic alignment total knee arthroplasty using augmented reality technology to maintain limb alignment within targeted boundaries

**DOI:** 10.1186/s42836-026-00371-0

**Published:** 2026-02-28

**Authors:** Sachiyuki Tsukada, Hiroyuki Ogawa, Tsutomu Nakayama, Shiho Minami, Masayoshi Saito, Naoyuki Hirasawa

**Affiliations:** 1 Department of Orthopaedic Surgery, Hokusuikai Kinen Hospital, 3-2-1 Higashihara, Mito, Ibaraki 310-0035 Japan; 2 Department of Rehabilitation, Hokusuikai Kinen Hospital, 3-2-1 Higashihara, Mito, Ibaraki 310-0035 Japan

**Keywords:** Knee replacement, Smartphone, Computer-assisted surgery, Augmented reality, Kinematic alignment

## Abstract

**Background:**

An augmented reality (AR)-aided navigation system that utilizes a standard smartphone enables accurate alignment of femoral and tibial components in total knee arthroplasty (TKA) and provides real-time intraoperative quantification of joint gaps. This study aimed to integrate the AR-aided navigation system into the surgical technique of restricted kinematic alignment and to evaluate its clinical effectiveness.

**Methods:**

We compared 45 restricted kinematic alignment TKAs performed using posterior cruciate ligament (PCL) retaining medial-congruent prosthesis with an AR-aided navigation system and 40 mechanically aligned TKAs performed using PCL resecting posterior-stabilized prosthesis in patients with preoperative varus or neutral lower limb alignment. In the restricted kinematic alignment group, femoral and tibial alignments were determined using calipered and soft tissue–guided techniques, respectively, with the AR-aided navigation system providing real-time confirmation that angular values remained within safe boundaries. The target intraoperative extension gap was a rectangular configuration with equal medial and lateral widths.

**Results:**

Intraoperative measured value of the soft-tissue imbalance with the knee in extension was significantly smaller in the restricted kinematic alignment group than in the mechanical alignment group (1.1 ± 1.1° vs 2.5 ± 2.2°; 95% CI, 0.7 to 2.2°; *P* < 0.001; Cohen’s d = 0.85). After propensity score matching, no significant differences were observed between the groups in either the timed up-and-go test or the 10-m walk test at 1 week postoperatively (12.6 ± 3.5 s vs 13.2 ± 5.3 s; 95% CI, − 3.0 to 1.7; *P* = 0.60; and 11.7 ± 2.5 s vs 11.9 ± 3.2 s; 95% CI, − 1.7 to 1.3; *P* = 0.82, respectively).

**Conclusions:**

Restricted kinematic alignment TKA using PCL retaining medial-congruent prosthesis achieved more balanced intraoperative soft-tissue tension than the mechanical alignment TKA using posterior-stabilized prosthesis. However, early postoperative walking speed did not differ between the two groups.

## Introduction

Mechanical alignment in total knee arthroplasty (TKA) aims to maximize implant longevity by equalizing the load on the medial and lateral compartments of the knee by aligning both femoral and tibial components perpendicular to the mechanical axis [1]. Nevertheless, kinematic alignment has attracted increasing interest, as it may offer several kinematic advantages over mechanical alignment, including greater femoral rollback [2] and a reduction in the adduction moment applied to the knee joint [3].

In kinematic alignment, the femoral component is typically placed in valgus and the tibial component in varus relative to the mechanical axis [1]. Excessive valgus positioning of the femoral component has been associated with an increased risk of reoperation due to anterior knee pain after kinematic alignment TKA [4], whereas excessive varus placement of the tibial component may be linked to higher rates of revision TKA [5]. The concept of restricted kinematic alignment has been proposed to mitigate these risks, in which defined boundaries are applied to the femoral valgus and tibial varus resection angles [6]. Maintaining resection angles within the defined boundaries is challenging to reproduce using conventional techniques, for which computer-assisted surgery is advantageous in restricted kinematic alignment [7]. Nevertheless, clear evidence is lacking to demonstrate the superiority of kinematic over mechanical alignment [8], and the exclusive use of costly computer systems cannot be justified for restricted kinematic alignment. There is an increasing need for a cost-effective system that can reliably support the kinematic alignment of TKA.

We developed the AR-KNEE system (Zimmer-Biomet Japan, Tokyo, Japan), which is a surgical navigation tool to achieve high cost-effectiveness by utilizing a standard consumer smartphone [9]. It has been reported to provide acceptable accuracy for femoral and tibial alignment in TKA [9, 10]. Although the AR-KNEE system is classified as a portable navigation system, it differs substantially from first-generation portable navigation systems in its ability to quantify the joint gap intraoperatively. We developed a surgical technique using the AR-KNEE system for restricted kinematic alignment, enabling precise control of the resection angles within the defined boundaries. The purpose of this study was to describe the surgical technique of the restricted kinematic alignment TKA using the AR-KNEE system and to compare the early radiographic and clinical outcomes between kinematic alignment-posterior cruciate ligament (PCL) retaining TKA with the mechanical alignment-PCL sacrificed TKA. This study did not aim to isolate alignment philosophy; rather, it compared two clinically implemented approaches differing in both alignment targets and implant/PCL strategy.

## Materials and methods

We retrospectively reviewed all patients who underwent TKA performed by a single surgeon (ST) at a single institution between June 2025 and October 2025. All procedures were performed without the use of a tourniquet. Surgical exposure of the knee joint was achieved through an anterolateral skin incision and a medial parapatellar approach. Both the femoral and tibial components were implanted using cement fixation. In patients with preoperative varus or neutral lower limb alignment, the Persona medial-congruent (MC) prosthesis (Zimmer Biomet, Warsaw, IN) was implanted according to the concept of restricted kinematic alignment when the AR-KNEE system was available. When the AR-KNEE system was not available, the Persona posterior-stabilized (PS) prosthesis (Zimmer Biomet) was implanted according to the concept of mechanical alignment. This allocation reflected limited AR-KNEE availability shortly after regulatory approval in Japan, permitting use of only one navigation unit per surgical day, with patient selection determined by the surgeon. Because the MC prosthesis was used with a PCL-retaining strategy, it was preferentially selected for patients considered to have a functional PCL, as suggested by preserved preoperative knee flexion. Regardless of the targeted lower limb alignment, the intraoperative extension gap was adjusted to achieve a rectangular configuration with equal medial and lateral widths. In all patients with preoperative valgus alignment, TKA was performed according to the concept of mechanical alignment.

The inclusion criterion was patients with preoperative varus or neutral lower limb alignment who received the Persona TKA prosthesis. Exclusion criteria were patients with valgus alignment, those who underwent unilateral TKA using a prosthesis other than Persona, and those who underwent simultaneous bilateral TKA.

We compared restricted kinematic alignment TKA using the Persona MC prosthesis with posterior cruciate ligament (PCL) retention and mechanical alignment TKA using the Persona PS prosthesis with PCL sacrifice.

### Surgical technique of the restricted kinematic alignment with AR-KNEE system

The AR-KNEE system was used to verify that both femoral and tibial resection angles remained within a boundary. No additional skin incision was required to employ the restricted kinematic alignment technique with the AR-KNEE system. All surgical procedures were completed by the surgeon in the sterile zone alone.

**Fig. 1:**
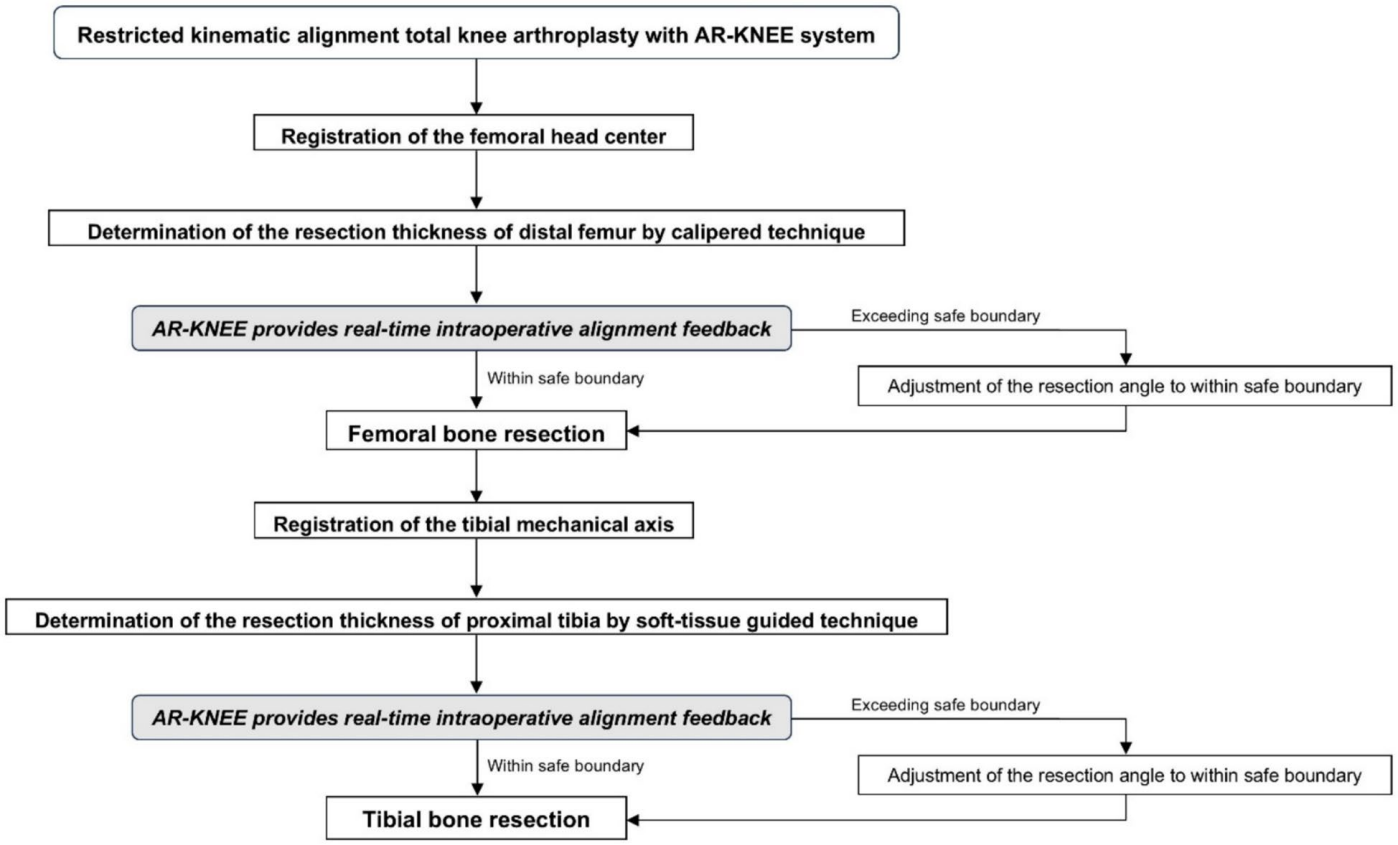
Workflow of restricted kinematic alignment TKA using the AR-KNEE system

**Fig. 2:**
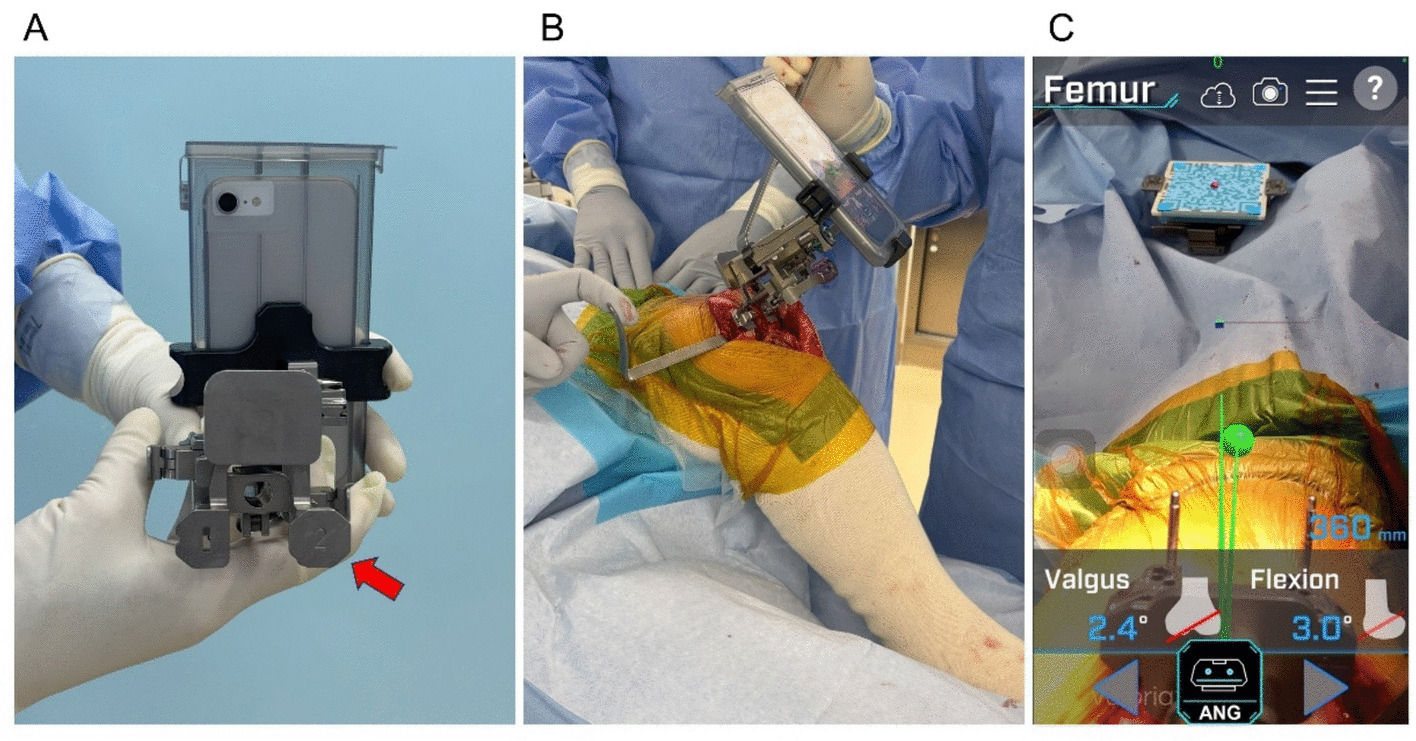
Distal femoral bone resection using the calipered technique aided by the AR-KNEE system. **A** A metal block shim with the same thickness as the cartilage defect of the distal medial femoral condyle (red arrow) was attached to the medial paddle of the distal cutting block of the AR-KNEE system. **B** Both the medial and lateral paddles of the distal cutting block were fitted to the distal femur. **C** Smartphone display of Fig. 2B. The bone resection angle after compensating for the medial cartilage defect was 2.4° in valgus for this patient. If the valgus angle exceeded the defined boundary, the surgeon could adjust the resection angle accordingly

**Fig. 3:**
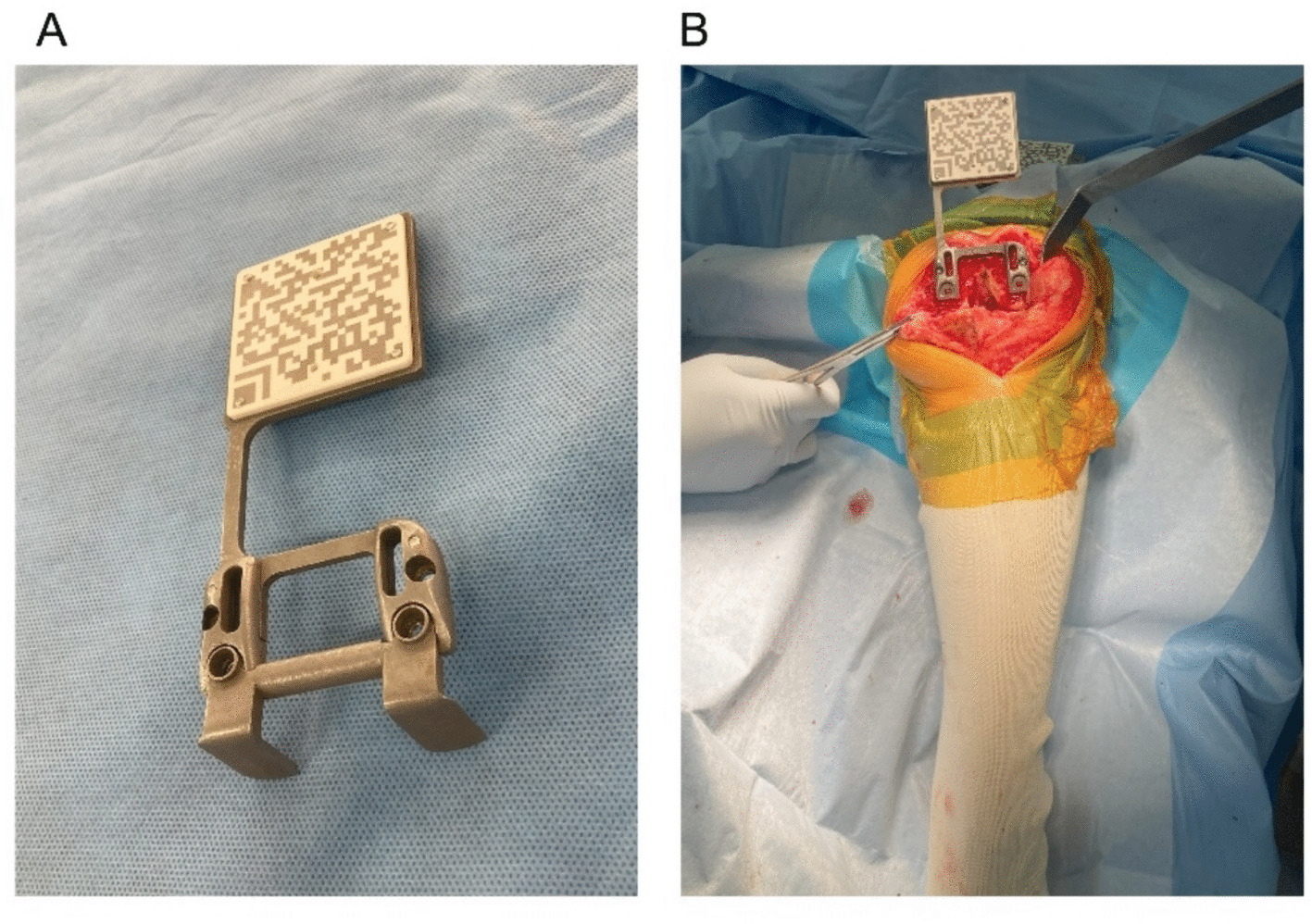
Plate with QR code marker for joint gap measurement in the AR-KNEE system. **A** Appearance of the plate with the QR code marker. The plate has detachable feet, which are removed after it is fixed to the resected surface of the distal femur. **B** The plate was positioned flush against the resected distal femoral surface, with its feet aligned against the posterior condyles. The feet were then removed. Note that no additional incision is required to assess the joint gap

Although kinematic alignment TKA aims to restore the patient’s pre-arthritic constitutional lower limb alignment, restricted kinematic alignment TKA limits bone resection angles within predefined safe boundaries in patients with atypical anatomy. The AR-KNEE system displays the bone resection angles anticipated with kinematic alignment TKA, enabling intraoperative adjustment when these angles exceed the safe boundaries. Figure 1 presents the workflow of the restricted kinematic alignment procedure using the AR-KNEE system. The distal femoral bone resection was performed using the calipered technique [11] with assistance from the AR-KNEE system. We first evaluated the thickness of the cartilage defect on the distal medial femoral condyle, with 2 mm being the most common defect size. To compensate for the defect, a corresponding metal block shim was attached to the medial paddle of the distal cutting block of the AR-KNEE system (Fig. 2A). For example, when the medial defect was measured as 2 mm, a 2-mm metal block shim was secured to the medial paddle. The keel of the AR-KNEE system was then inserted into the distal femur, and the distal cutting block with the mounted smartphone was attached to the keel [10]. After registering the femoral head center according to the standard AR-KNEE surgical procedure, the paddle of the distal cutting block equipped with the compensatory metal block shim was aligned with the distal femur (Fig. 2B). The AR-KNEE system displayed the difference between the distal cutting angle and the femoral mechanical axis (Fig. 2C). In cases where the distal cutting angle exceeded the valgus boundary defined by the surgeon (5°), the cutting angle was adjusted to 5° of valgus. The plate with the QR code marker was placed on the resection surface of the distal femur, aligned parallel to the posterior condylar axis in the axial plane. (Fig. 3).

**Fig. 4:**
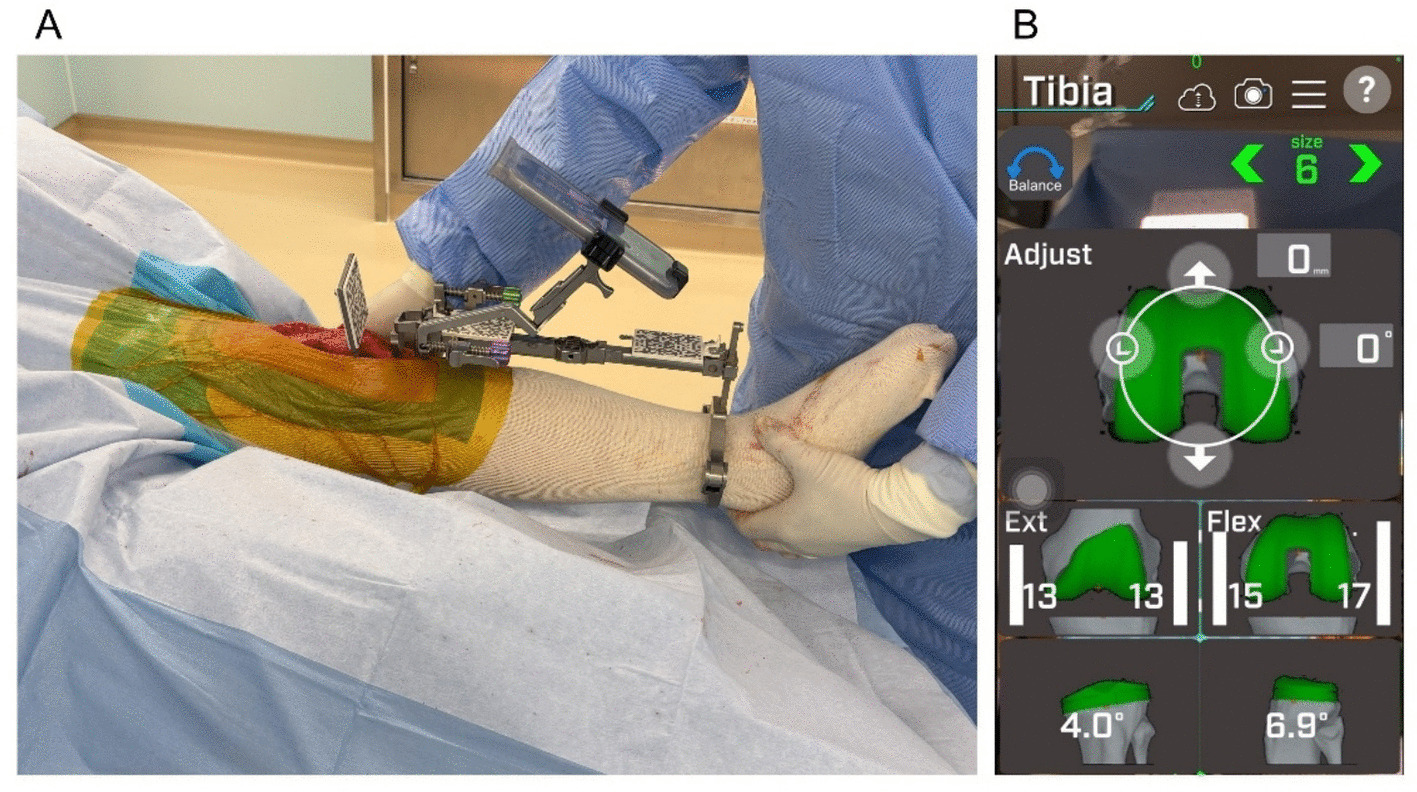
Proximal tibial bone resection using the soft tissue-guided technique aided by the AR-KNEE system. **A** Manual in-line traction of the knee joint. Longitudinal distraction aligned the joint according to the balance of medial and lateral soft tissue tension. **B** Smartphone display corresponding to Fig. 4A. The AR-KNEE system indicated that the medial and lateral joint gaps in extension would be equal (13 mm) if the proximal tibia were resected at 4.0° varus and a 6.9° posterior slope. If the varus angle displayed by the navigation system exceeded the surgeon-defined boundary, the resection angle could be adjusted

The proximal tibial cutting angle and thickness were determined using the soft tissue-guided technique [12] with support from the AR-KNEE system. This approach was chosen because the proximal tibia is markedly deformed in most patients undergoing TKA, making it difficult to predict the tibial resection line to reconstruct the native joint line based on the morphology of the proximal tibia during surgery. First, the surgeon fixed the tibial cutting guide with QR-code markers to the lower limb, and registered the anatomical landmarks to establish a 3-dimensional coordinate system of the lower limb. Next, the surgeon applied continuous longitudinal distraction to the knee joint (Fig. 4A). This manual distraction aligned the knee joint according to the balance between medial and lateral soft tissue tension due to the ligamentotaxis theory. During the in-line traction, the surgeon visually confirmed that both the femur and tibia were free from internal or external rotational malalignment. The distraction was maintained until the medial collateral ligament was fully tensioned. The smartphone displayed the medial and lateral joint gap widths at that time. Based on these values, the surgeon adjusted the proximal tibial resection angle in the coronal plane to equalize the medial and lateral joint gaps (Fig. 4B). In cases where the proximal cutting angle exceeded the varus boundary defined by the surgeon (5°), the cutting angle was adjusted to 5° of varus. The targeted tibial posterior slope was set at 7° for all patients in the restricted kinematic alignment group.

The femoral rotation angle was parallel to the posterior condylar line. The tibial rotation angle was aimed to be parallel to Akagi’s anteroposterior line with the aid of the AR-KNEE system [13]. No soft tissue release was performed to adjust the soft tissue balance.

### Surgical technique of the control group

In the control group, all procedures were performed by the same surgeon as in the restricted kinematic alignment group, in accordance with the mechanical alignment principle using a Persona PS prosthesis without computer assistance [14]. The femoral component was aligned parallel to the surgical epicondylar axis, and the tibial component was aligned parallel to Akagi’s anteroposterior line. The surgeon endeavored to minimize soft tissue release, performing only limited releases necessary for osteophyte removal [14]. When medial soft tissue tension was deemed excessively tight during soft tissue balance assessment, the surgeon addressed it by performing an additional varus resection of the proximal tibia rather than further soft tissue release.

### Quantifying the soft tissue balance

Immediately before cementing the prostheses, intraoperative soft tissue balance was quantified using an offset-type TKA tensor (Offset Repo-Tensor; Zimmer-Biomet) with the femoral component in place and with the patellofemoral joint reduced [15]. The Offset Repo-Tensor has two plates: an upper seesaw plate and a lower platform plate [15]. The joint center gap (millimeter) and varus ligament balance (degree) between the two plates were recorded using the tensor with a distraction force of 30 pounds [14].

### Measuring the lower limb alignment

We evaluated preoperative and postoperative standing long-leg radiographs obtained with the patella oriented anteriorly. Postoperative radiographs were acquired 2 weeks after TKA. The lateral distal femoral angle (LDFA) and medial proximal tibial angle (MPTA) were measured, and the arithmetic hip–knee–ankle (aHKA) angle and joint line obliquity (JLO) were calculated using the formulas described by MacDessi et al. [16].

### Outcome measures

The primary outcome was the intraoperative soft-tissue balance measured with the knee in extension. The Offset Knee Balancer quantifies varus–valgus imbalance based on the tilting angle between its two plates [15]. When the tilting angle indicated greater medial than lateral soft-tissue tension, the deviation was classified as varus and assigned a negative value [17]. Conversely, when lateral soft-tissue tension exceeded medial tension, the deviation was classified as valgus and assigned a positive value [17]. The secondary outcomes include timed-up-and go test [18] and the 10-m walk test [19].

### Sample size calculation and statistical analysis

All statistical analyses were implemented with the use of the R statistical package (R Foundation for Statistical Computing).

Prior to initiating the study, we determined a 1° difference in soft tissue varus–valgus imbalance as clinically meaningful in comparison of the two target alignments in TKA. The sample size was calculated to detect this difference between groups. A total of 36 patients per group was required to achieve 80% power to detect a 1° difference using a two-sided Student *t*-test with an α level of 0.05. A standard deviation of 1.5° was assumed for the calculation based on preliminary data. For the primary outcome, we compared the 2 groups with use of the Student’s *t*-test and reported 95% confidence intervals for the between-group difference. The magnitude of the observed differences was quantified using Cohen’s d, with effect sizes of 0.2–0.5 considered small, 0.5–0.8 moderate, and ≥ 0.8 large.

For baseline characteristics and secondary outcomes, continuous variables were reported as means and standard deviations and compared using the Student’s *t*-test. Categorical variables were presented as counts and compared using Fisher’s exact test. Because early postoperative ambulatory function is substantially influenced by preoperative walking ability [20], propensity score matching was performed when comparing the timed up-and-go test and 10-m walk test between the two groups. Propensity scores were calculated using age, sex, body mass index, and preoperative 10-m walk test performance as covariates. Matching was conducted using a nearest-neighbor algorithm with a caliper width of 0.2 times the standard deviation, applying a 1:1 matching ratio. Covariate balance after matching was evaluated using standardized mean differences. A standardized mean difference 10% or less than 10% was considered to indicate adequate balance, whereas a standardized mean difference exceeding 25% was considered indicative of substantial imbalance.

## Results

During the study period, 108 patients underwent TKA. Of these, 6 patients were excluded due to valgus preoperative limb alignment, 5 patients were excluded because a prosthesis other than Persona was selected, and 12 patients were excluded for having undergone simultaneous bilateral TKA. The remaining 85 patients met the inclusion criteria for this study. Among them, 45 patients underwent TKA of the restricted kinematic alignment with the AR-KNEE system, and 40 patients underwent TKA of the conventional mechanical alignment.

The preoperative characteristics of the two groups are presented in Table 1. There were no significant differences between the groups in any preoperative lower limb alignment parameters. The preoperative knee flexion angle was significantly better in the restricted kinematic alignment group. Operative time was significantly longer in the restricted kinematic alignment group.

**Table 1:** Patient demographic and baseline clinical characteristics

	**Restricted kinematic alignment (*n* = 45)**	**Mechanical alignment (*n* = 40)**	***P*-value**	**Standardized mean difference**
Age, years	76 ± 6	75 ± 8	0.11*	0.357
Sex (female/male)	39/6	29/11	0.74†	0.071
Height, cm	152 ± 8	153 ± 9	0.59*	0.119
Weight, kg	61.8 ± 11.1	65.6 ± 16.5	0.21*	0.272
Body mass index, kg/m^2^	26.6 ± 3.9	27.7 ± 4.8	0.25*	0.249
Preoperative lateral distal femoral angle, °	88.9 ± 2.1	88.7 ± 2.9	0.70*	0.084
Preoperative medial proximal tibial angle, °	83.9 ± 2.4	83.2 ± 3.4	0.28*	0.233
Preoperative arithmetic hip-knee-ankle angle, °	− 5.0 ± 3.2	− 5.5 ± 4.9	0.59*	0.116
Preoperative joint line obliquity, °	172.8 ± 3.1	171.9 ± 4.1	0.25*	0.25
Preoperative knee flexion angle, °	122 ± 16	113 ± 20	0.04*	0.463
Preoperative knee extension angle, °	− 7 ± 4	− 9 ± 6	0.076*	0.399
Preoperative timed up-and-go test, second	10.6 ± 4.2	12.8 ± 6.4	0.069*	0.396
Preoperative 10-m walk test, second	10.4 ± 3.3	12.4 ± 5.4	0.039*	0.45
Operative time, minute	74 ± 6	67 ± 8	< 0.001*	1.068

Results are expressed as means ± standard deviation, unless otherwise indicated

^*^ *P*-values were determined with Student’s *t-test*

^†^ *P*-values were determined with a chi-squared test

### Postoperative lower limb alignment

The postoperative LDFA was significantly smaller in the restricted kinematic alignment group (88.8 ± 1.6° versus 90.7 ± 1.6°, *P* < 0.001), indicating that the femoral component was implanted in greater valgus. There was no significant difference between the groups in the postoperative MPTA (88.2 ± 1.9° versus 88.9 ± 2.0°, *P* = 0.097).

The postoperative aHKA angle was significantly larger in the restricted kinematic alignment group (− 0.5 ± 2.5° versus − 1.8 ± 2.4°, *P* = 0.022), indicating that postoperative lower limb alignment was more varus in the mechanical alignment group. The postoperative JLO was significantly smaller in the restricted kinematic alignment group (177.0 ± 2.5° versus 179.7 ± 2.6°, *P* < 0.001), indicating a more medially inclined joint line.

### Primary and secondary outcomes

The intraoperative varus/valgus soft-tissue imbalance in knee extension was significantly smaller in the restricted kinematic alignment group than in the mechanical alignment group (1.1 ± 1.1° vs 2.5 ± 2.2°; 95% CI, 0.7–2.2°; *P* < 0.001), with a large effect size (Cohen’s d = 0.85).

**Fig. 5:**
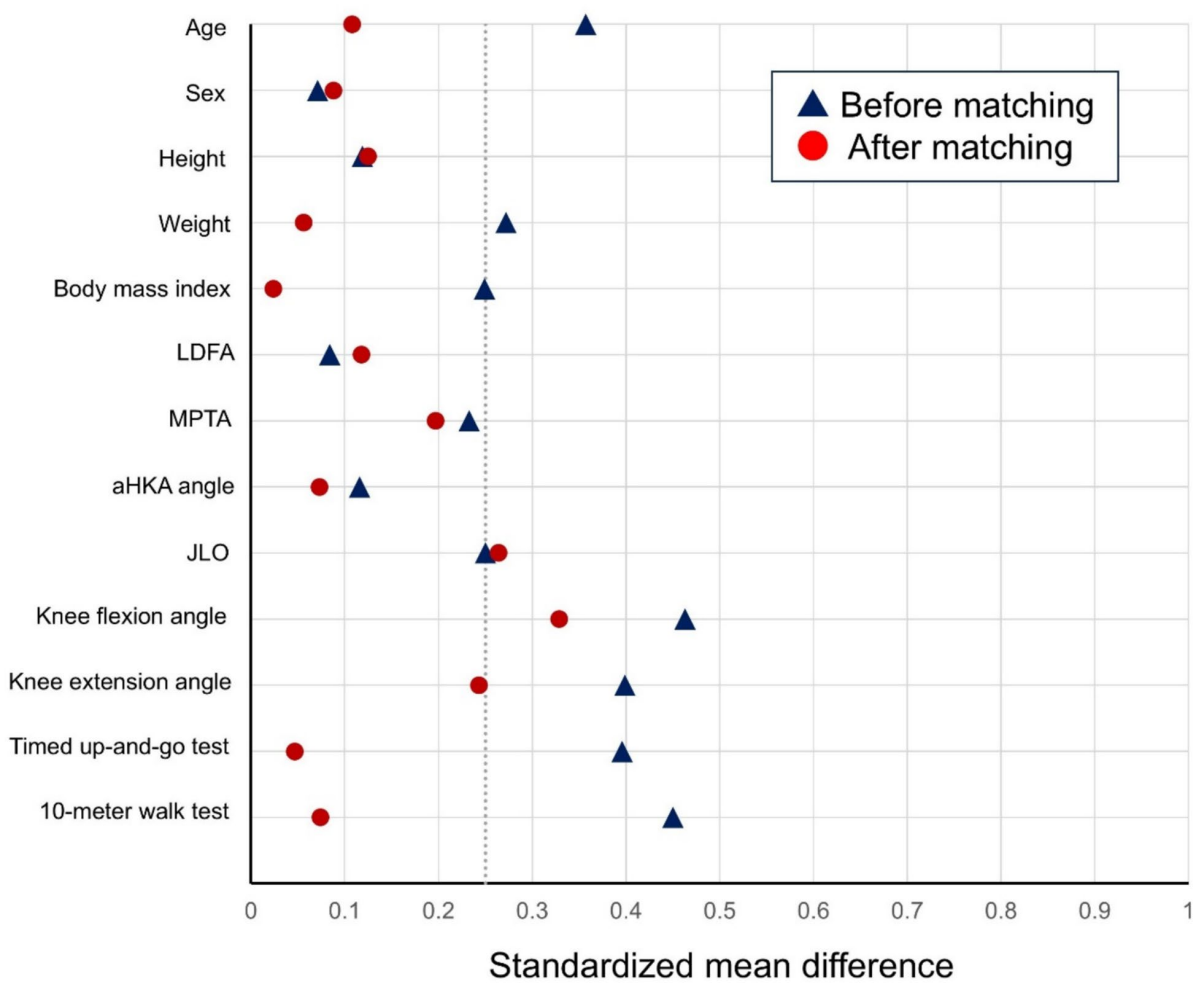
Love plot showing the standardized mean difference before and after propensity score-matching using age, sex, body mass index, and preoperative 10-m walk test as covariates. *LDFA, lateral distal femoral angle; MPTA, medial proximal tibial angle; aHKA, arithmetic hip–knee–ankle; JLO, joint line obliquity*

We matched 29 patients of the restricted kinematic alignment group with those of the mechanical alignment group (Table 2). Figure 5 presents the Love plot illustrating the standardized mean differences of preoperative patient characteristics before and after matching. The matching procedure only partially reduced baseline imbalances; specifically, the standardized mean differences for preoperative JLO and knee flexion angle remained greater than 25%, indicating substantial residual imbalance. After matching, there were no difference between the two groups in both the timed up-and-go test and 10-m walk test 1 week after TKA (12.6 ± 3.5 s versus 13.2 ± 5.3 s; 95% CI, − 3.0 to 1.7; *P* = 0.60, and 11.7 ± 2.5 s versus 11.9 ± 3.2 s; 95% CI, − 1.7 to 1.3; *P* = 0.82, respectively).

**Table 2:** Patient demographic and baseline clinical characteristics after propensity score matching

	**Restricted kinematic alignment (*n* = 29)**	**Mechanical alignment (*n* = 29)**	***P-*value**	**Standardized mean difference**
Age, years	75 ± 6	75 ± 8	0.68*	0.108
Sex (female/male)	24/5	23/6	> 0.99†	0.088
Height, cm	154 ± 9	153 ± 9	0.64*	0.125
Weight, kg	63.3 ± 12.7	62.6 ± 13.0	0.83*	0.056
Body mass index, kg/m^2^	26.7 ± 4.2	26.8 ± 4.6	0.93*	0.024
Preoperative lateral distal femoral angle, °	88.9 ± 2.3	88.6 ± 2.4	0.66*	0.118
Preoperative medial proximal tibial angle, °	83.9 ± 2.4	83.3 ± 3.5	0.46*	0.197
Preoperative arithmetic hip-knee-ankle angle, °	− 5.0 ± 3.3	− 5.3 ± 5.0	0.78*	0.073
Preoperative joint line obliquity, °	172.8 ± 3.3	171.9 ± 3.2	0.32*	0.264
Preoperative knee flexion angle, °	122 ± 15	116 ± 19	0.22*	0.329
Preoperative knee extension angle, °	− 7 ± 5	− 8 ± 5	0.37*	0.243
Preoperative timed up-and-go test, second	10.9 ± 4.0	11.1 ± 4.7	0.86*	0.047
Preoperative 10-m walk test, second	10.3 ± 2.8	10.6 ± 3.4	0.89*	0.074
Operative time, minute	75 ± 5	66 ± 8	< 0.001*	1.415

Results are expressed as means ± standard deviation, unless otherwise indicated

^*^ *P*-values were determined with Student’s *t-test*

^†^ *P*-values were determined with a chi-squared test

## Discussion

The intraoperative extension gap imbalance was smaller in the restricted kinematic alignment TKA using a Persona MC prosthesis with PCL retention than in the mechanical alignment TKA using a Persona PS prosthesis. However, no significant difference in walking speed was observed between the two groups at one week postoperatively.

The AR-KNEE system is a relatively low-cost navigation system as it works with the use of a smartphone and resin markers with QR code [9, 10]. The cost-effective AR navigation system may represent an important option for facilitating restricted kinematic alignment. As the restricted kinematic alignment procedure requires highly precise adjustment of bony resection angles, accuracy may not be consistently attainable with manual techniques alone. Consequently, the need for computer-assisted surgery to safely perform restricted kinematic alignment has been emphasized since the early stages of its development [7]. The use of costly navigation technologies for restricted kinematic alignment procedures has been debated because the clinical utility of kinematic alignment remains controversial [8, 21]. Thus, there is a growing demand for an affordable system capable of supporting the technique reliably. Furthermore, the AR-KNEE system offers additional advantages, including the elimination of supplementary pin insertions, thereby reducing the risk of major complications such as femoral fracture or pin-site infection.

The restricted kinematic alignment group exhibited more balanced mediolateral soft-tissue tension in extension than the mechanical alignment group. However, the present study was not designed to directly contrast the alignment philosophies of restricted kinematic alignment and mechanical alignment, as differences in prosthesis design and PCL management undoubtedly influenced the outcomes. Restricted kinematic alignment TKA was performed using a PCL-retaining Persona MC prosthesis, whereas mechanical alignment TKA employed a PCL-resecting PS prosthesis; these prosthesis types differ fundamentally in constraint, femoral rollback characteristics, and their effects on intraoperative gap behavior. Furthermore, extension gap adjustment strategies differed between groups: in the restricted kinematic alignment group, the extension gap was established through soft tissue–guided tibial resection, whereas in the mechanical alignment group, it was adjusted by progressively increasing varus resection of the proximal tibia to avoid medial soft-tissue release. Consequently, the relative contributions of alignment strategy, prosthesis design, and PCL management to the observed outcomes cannot be determined from this study alone.

The mean value of the parameters for the lower limb alignments in the restricted kinematic alignment group demonstrated a valgus femoral component position, varus tibial component orientation, a varus overall limb alignment, and a medially inclined joint line as commonly observed in kinematic alignment [1]. In this study cohort, the mechanical alignment group exhibited even greater tibial varus positioning, likely reflecting a surgical preference to balance soft tissues primarily by altering proximal tibial resection angle rather than performing additional medial soft-tissue release. Bone recut adjustment techniques have increasingly been adopted to minimize medial soft-tissue release in PS TKA in recent practice [22]. Accordingly, the findings of the present study should be interpreted with caution, as these techniques and the resulting postoperative alignment deviate from conventional mechanical alignment philosophy and may have influenced the observed outcomes.

This study has several limitations. First, the retrospective design may introduce selection bias. In this cohort, patients who underwent PCL-preserving procedures demonstrated significantly greater preoperative knee flexion, likely reflecting patient selection at the surgeon’s discretion. Second, the comparison was between restricted kinematic alignment TKA performed with an MC prosthesis and PCL preservation, and mechanical alignment TKA performed with a PS prosthesis and PCL resection. Therefore, differences in PCL management and prosthesis design likely affected the outcomes, and the results should not be interpreted as a direct comparison of alignment philosophies alone. Nevertheless, because evidence supporting kinematic alignment in PS-type TKA is limited and mechanical alignment may be more appropriate for PS prostheses [24], the current comparison may be more clinically meaningful than studies simply evaluating alignment strategies. Third, the sample size calculation was based on the primary outcome; therefore, the study may have been underpowered to detect differences in two tests assessing walking speed after TKA. Fourth, although propensity score matching was employed to reduce baseline imbalances, complete covariate balance was not achieved; therefore, comparisons of postoperative walking speed between the groups should be interpreted with limited confidence. Finally, standing long-leg radiographs were obtained 2 weeks after TKA, which may be too early to accurately assess the lower limb alignment because postoperative swelling and patient guarding can influence weight-bearing radiographic measurements.

## Conclusions

The AR-KNEE system provides a cost-effective navigation solution enabling accurate restricted kinematic alignment through precise control of bone resection and soft-tissue balance. Using the restricted kinematic alignment TKA with Persona MC prosthesis and PCL retention resulted in more balanced extension gaps than mechanical alignment TKA with Persona PS prosthesis, although early postoperative walking performance was similar between groups.

## Data Availability

The datasets used and/or analyzed during the current study are not publicly available. Data are, however, available from the corresponding author on reasonable request.
